# Managing established bronchopulmonary dysplasia without using routine blood gas measurements

**DOI:** 10.1038/s41372-024-01955-x

**Published:** 2024-04-23

**Authors:** Matthew J. Kielt, Laurie C. Eldredge, Edward G. Shepherd, Robert J. DiGeronimo, Audrey N. Miller, Roopali Bapat, George El-Ferzli, Stephen E. Welty, Leif D. Nelin

**Affiliations:** 1 Comprehensive Center for Bronchopulmonary Dysplasia, Nationwide Children’s Hospital and Department of Pediatrics, The Ohio State University, Columbus, OH USA; 2 The BPD Program at Seattle Children’s Hospital and the Department of Pediatrics, University of Washington, Seattle, WA USA

**Keywords:** Paediatrics, Respiratory tract diseases

## Abstract

**Objective:**

Routine blood gas measurements are common in infants with severe bronchopulmonary dysplasia (sBPD) and are a noxious stimulus. We developed a guideline-driven approach to evaluate the care of infants with sBPD without routine blood gas sampling in the chronic phase of NICU care (after diagnosis at 36 weeks PMA).

**Study design:**

We examined blood gas utilization and outcomes in our sBPD inpatient care unit using data collected between 2014 and 2020.

**Results:**

485 sBPD infants met inclusion criteria, and 303 (62%) never had a blood gas obtained after 36 weeks PMA. In infants who had blood gas measurements, the median number of total blood gases per patient was only 4 (IQR 1–10). We did not identify adverse effects on hospital outcomes in patients without routine blood gas measurements.

**Conclusions:**

We found that patients with established BPD could be managed without routine blood gas analyses after 36 weeks PMA.

## Introduction

Bronchopulmonary dysplasia (BPD) is the most common complication of preterm birth, affecting approximately 50% of infants born < 30 weeks gestation [1, 2]. BPD is defined as a need for respiratory support at 36 weeks post-menstrual age (PMA) in infants born at < 32 weeks gestation [3]. Severity of disease is described by 3 grades, where grades 2 and 3 are considered severe BPD due to the need for positive pressure respiratory support at 36 weeks PMA [3]. Advances in neonatal intensive care have improved survival in very low birthweight (VLBW) and extremely preterm (EP) infants [4, 5]. However, as survival has increased, the rates of BPD have also increased [1]. These increases have occurred despite the development of multiple interventions that have been associated with reduced risk of BPD, including non-invasive respiratory support, surfactant, gentle invasive ventilation, and skin-to-skin care [6–8].

BPD is associated with increased mortality and long-term morbidities, including neurodevelopmental impairment (NDI) [9, 10]. The mechanisms underlying the association between BPD and NDI are controversial, but may include exposure to inflammation, hypoxia, infection, and illness severity [11, 12]. Additionally, infants with BPD have longer neonatal intensive care unit (NICU) length of stay (LOS) and are exposed to greater numbers of noxious stimuli, more neurosedative medications, and fewer positive interactions than those without BPD [13]. Typical NICU patients endure significant numbers of painful procedures [14] and recent evidence suggests that exposure to noxious stimuli is independently associated with NDI [15]. Contemporary chronic care models in the NICU for severe BPD emphasize reducing exposure to noxious stimuli to reduce the risk of NDI [16–18]. The most frequent noxious stimuli that preterm infants experience is the heel lance, often to obtain blood gases [17], and some studies in preterm infants suggest that blood gas sampling is painful, costly, and potentially inaccurate [19, 20]. However, routine blood gas sampling is a common NICU practice with some centers reporting universal routine blood gas screening of patients with BPD after 36 weeks PMA [21, 22].

We have developed a multidisciplinary guidelines-driven treatment approach built on the principles of chronic care for patients with established severe BPD that emphasizes: (1) chronic phase ventilation, (2) nutrition, and (3) optimizing neurodevelopment by minimizing exposures that interfere with brain development [17, 18]. One way to minimize noxious stimuli that we have adopted is elimination of routine blood gas measurements after 36 weeks PMA. There are no accepted standards for when a blood gas measurement is clinically indicated in established severe BPD, therefore blood gases are ordered for clinical indications at the discretion of the attending physician in our BPD NICU. In our BPD NICU these clinical indications may involve an unexpected clinical change, prolonged non-response to therapies, and/or at the request of sub-specialty consultants, but there are no established protocols or guidelines in our BPD NICU for obtaining clinically indictated blood gases. The objective of this observational study was to (1) determine how the elimination of routine blood gas measurements has affected our use of “clinically indicated” blood gases in infants with severe BPD after admission to our BPD NICU and (2) to compare clinical outcomes in sBPD infants without routine blood gas measurements to those with clinically indicated blood gases.

## Methods

### Study population

The Nationwide Children’s Hospital (NCH) institutional review board approved this study with a waiver of consent. We performed an observational study of infants admitted to the BPD intensive care unit at NCH [10, 17, 18]. We included infants with established BPD as defined by the NICHD Neonatal Research Network [3] who were admitted between January 1, 2014 and May 30, 2020. We excluded infants with major congenital malformations or genetic syndromes. Data gathered from study subjects included demographics, birth characteristics, and clinical characteristics at 36 weeks PMA and at discharge.

### Study location

The BPD NICU at NCH is a specialized 24-bed referral unit for infants with BPD. Patients are referred to our BPD NICU after the diagnosis of BPD is made at 36 weeks PMA. Patient care in the BPD NICU is informed by a guideline-driven multidisciplinary approach that emphasizes chronic phase ventilation, nutrition, and neurodevelopment care that includes minimizing exposures to noxious stimuli [10, 17, 18]. As such, our practice has evolved to eliminate routine blood gas measurements in patients with established BPD, even in those patients supported with invasive mechanical ventilation or non-invasive positive airway pressure (i.e., nasal constant positive airway pressure (CPAP), high-flow nasal cannula, non-invasive positive pressure ventilation (nIPPV)). In place of routine blood gas measurements, we assess the respiratory status of our BPD patients using serial physical exam assessments (specifically chest rise, breath sounds, and baseline work of breathing), growth parameters, tolerance of developmental therapies, and FiO_2_ requirements. Although, we rely primarily on these assessments, other non-invasive assessments are sometimes also used including but not limited to ventilator data (such as tidal volumes, flow-volume loops, flow scalars, etc.), respiratory severity score, and SpO2/FiO2 ratio. Respiratory support is adjusted if needed based on these non-invasive evaluations [18]. Our guidelines permit blood gas sampling for clinical indications at the discretion of the attending physician.

### Study outcomes

We assessed the number of blood gas measurements done in our population after 36 weeks PMA. We also assessed the number of blood gases per patient for the entire hospitalization in the BPD NICU and the cost of blood gas measurements on the patients that had blood gases done. We also examined in-hospital outcomes including mortality, tracheostomy, and death or tracheostomy. The discharge status of survivors was recorded including LOS, PMA at discharge, discharged on room air, supplemental oxygen, positive pressure, and/or gastrostomy.

We also compared our clinical outcomes with other cohorts of BPD patients wherein care practices included blood gas sampling; we identified published cohorts that also included similar outcome metrics. Interestingly, there are a relative paucity of published cohorts of infants with established BPD. Most larger cohort studies in neonatology related to BPD have been studies on preterm infants to examine the factors associated with the development of BPD, given that these cohorts include both patients with and without BPD these data are difficult to compare to our data. However, we did find four recent publications [2, 3, 23, 24] that described similar outcomes in cohorts of infants with established BPD. Finally, to compare our results in a cohort of infants with established BPD who had no blood gases done to a cohort of infants who had routine blood gas sampling, we identified two studies [22, 25] in infants with established BPD wherein routine blood gas sampling was specified.

### Statistical analysis

We analyzed descriptive statistics for demographics and clinical characteristics at 36 weeks PMA and at discharge using median and interquartile ranges [IQR] for continuous variables and number and percentages for categorical variables. The Mann-Whitney rank-sum test was used to compare continuous data and the chi-square test was used to compare categorical variables. The cost of blood gas studies were determined via the laboratory billing department. Statistical analysis was performed using R version 4.1.2 (R Foundation for Statistical Computing, Vienna, Austria). All *p*-values are two-tailed and considered significant if *p* < 0.05.

## Results

**Fig. 1:**
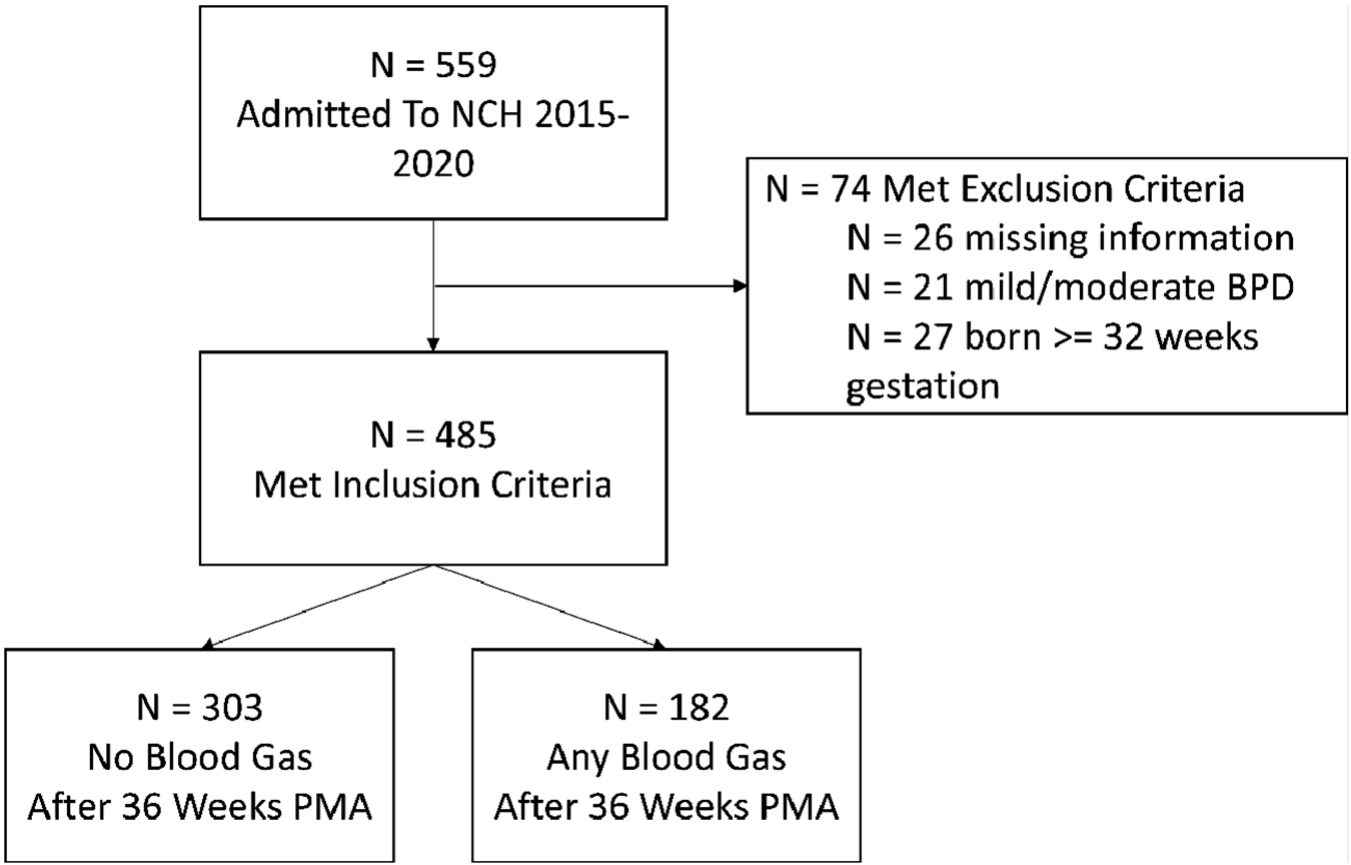
Flow diagram for the study. Five hundred and fifty-nine patients were admitted to the BPD NICU between 2015 and 2020 at ≥36 weeks PMA. Of those, 74 met exclusion criteria, leaving 485 that met inclusion criteria. There were 303 patients who never had a blood gas performed after admission to the BPD ICU.

A total of 485 infants with BPD met inclusion criteria (Fig. 1). Following our guidelines to eliminate routine blood gas sampling, 303 (62% of the cohort) never had a blood gas obtained after admission to the BPD NICU. The remaining 182 (38%) infants had at least 1 clinically indicated blood gas obtained after admission to the BPD NICU. In those 182 infants with at least one clinically indicated blood gas, the median number of total blood gases obtained throughout the BPD unit hospitalization per infant was only 4, with a significant left skew of the data as demonstrated by the IQR of 1–10. In those patients that had at least one clinically indicated blood gas drawn the total additional cost of blood gas sampling was $247,860, or an average total cost for blood gases of $1362 per patient.

As expected, this cohort of infants with established BPD had relatively severe illness. They were born extremely preterm, with a median gestational age (GA) of 26 weeks [24–27 weeks] and with extremely low birth weights (BW), with a median BW of 770 grams [615–975 grams] (Table 1). Most of the cohort received antenatal steroids, were delivered by cesarean section, were intubated in the delivery room, and received postnatal surfactant (Table 1). Most patients were admitted to the Level IV NICU at NCH before reaching 36 weeks PMA (Table 1); and were then transferred to the BPD NICU at time of diagnosis of BPD at or after 36 weeks PMA. Grade 3 BPD was diagnosed in 110 infants (23%).

**Table 1:** Demographics and neonatal characteristics of cohort.

Number	*485*
Maternal age (years)	29 (24–33)
Maternal Ethnicity - Hispanic or Latino	19 (4)
Maternal race	
American Indian or Alaskan native	1 (0.2)
Asian	14 (3)
Black	144 (30)
Native Hawaiian or pacific islander	1 (0.2)
White	307 (63)
Other	18 (4)
Complete antenatal steroids	319/400 (80)
Maternal chorioamnionitis	87 (18)
Cesarean delivery	347 (72)
Male	299 (62)
Gestational age (weeks)	26 (24–27)
Birth weight (grams)	770 (615–975)
Birth Length (cm)	32 (30–35)
Birth head circumference (cm)	23 (22–25)
Small for gestational age	88 (19)
Intubated in delivery room	335 (69)
Surfactant doses	2 (1-2)
PMA on admission to NCH (weeks)	29 (27–35)
Grade 3 BPD	110 (23)
No blood gases after 36 weeks PMA	303 (62)

Data shown as median [IQR] or *n* (%).

*PMA* post-menstrual age, *NCH* Nationwide Children’s Hospital.

Outcomes for the cohort are given in Table 2. The relative disease severity in this cohort is also demonstrated by mortality rate, tracheostomy rate, and the combined outcome death or tracheostomy (Table 2). In this cohort, 20% of survivors were discharged on room air, while nearly 70% of the infants were discharged on supplement oxygen and 3% were on positive pressure at discharge (Table 2). The median LOS was 125 days [IQR, 90–188] with a median PMA of 47 weeks [IQR, 42–56] at discharge (Table 2).

**Table 2:** Outcomes.

*Number*	*485*
Mortality	21 (4)
Death or tracheostomy	65 (13)
*Survivors at discharge*	*464*
Room air without respiratory support	93 (20)
Length of stay (days)	125 (90–188)
PMA at discharge (weeks)	47 (42–56)
Only supplemental oxygen	322 (69)
Tracheostomy	46 (10)
Gastrostomy	163 (35)
Positive pressure support	21 (3)

Data shown as median (IQR) or *n* (%).

*PMA* post-menstrual age, positive pressure support includes constant positive airway pressure (CPAP),

CPAP/pressure support (PS), high-flow nasal cannula (> 2 lpm) and mechanical ventilation.

We examined differences in demographics and clinical characteristics between the infants in this cohort that never had a blood gas measurement and those infants that had at least one clinically indicated blood gas measurement as shown in Table 3. The infants that had at least one blood gas obtained may have had greater disease severity than those infants who never had a blood gas done, as suggested by the birth weight, proportion that were small for gestational age (SGA), and the proportion with grade 3 BPD (Table 3). The outcomes were also consistent with the idea that the infants that had at least one blood gas done were relatively sicker than those that never had a blood gas done as demonstrated by group differences in mortality, tracheostomy, length of stay, and PMA at discharge (Table 3).

**Table 3:** Comparison of Patients who never had a blood gas with those that had at least one blood gas.

	Never Blood Gas after 36 weeks PMA	≥ 1 Blood Gas after 36 weeks PMA	
Number	*303*	*182*	–
***Demographics/Clinical data***			*p-value*
Maternal age (years)	29 [24–33]	28 [24–33]	0.9
Complete prenatal steroids	205/253 (81)	114/147 (78)	0.5
Maternal chorioamnionitis	53 (17)	34 (19)	0.8
Cesarean delivery	219 (72)	128 (70)	0.7
Male	183 (60)	116 (64)	0.4
Gestational age (weeks)	26 [24–27]	25 [24–27]	0.2
Birth weight (grams)	790 [630–990]	747 [586–930]	**0.02**
Birth length (cm)	33 [30–36]	32 [30–35]	0.09
Birth head circumference (cm)	23 [22–25]	23 [22–24]	0.2
Small for gestational age	46 (16)	42 (23)	**0.05**
Intubated in delivery room	202 (67)	133 (73)	0.2
Surfactant doses	2 [1,2]	2 [1,2]	0.2
PMA on Admission to NCH (weeks)	30 [27–35]	28 [26–34]	0.4
Grade 3 BPD	47 (16)	63 (35)	**< 0.001**
***Outcomes***			*Unadjusted between group difference (%, 95% CI)*
Mortality	2 (0.7)	19 (10)	10 (5–14)
Tracheostomy	18 (6)	28 (17)	11 (5–18)
Length of stay	109 [81–145]	168 [121–231]	77 (74–80)
PMA at discharge	45 [41–52]	54 [46–64]	11 (9–27)

Data shown as median [IQR] or *n* (%).

*PMA* post-menstrual age, *NCH* Nationwide Children’s Hospital.

As shown in Table 4, there were no fundamental differences in mortality, PMA at discharge, LOS, tracheostomy, or Grade 3 BPD compared to 4 other cohorts of patients with BPD described by the Children’s Hospitals Neonatal Consortium (CHNC), the NICHD Neonatal Research Network (NRN), the Vermont Oxford Network (VON), and the BPD Collaborative [2, 3, 23, 24]. It should be remembered that NCH is part of these networks. Finally, as shown in Table 5, our LOS, PMA at discharge, and duration of invasive mechanical ventilation were similar to 2 published cohorts of infants that specified routine blood gas sampling [22, 25].

**Table 4:** Comparison of our entire cohort to published large BPD cohorts.

Reference	*Lagatta* [23]	*Jensen*^*1*^ [3]	*Jensen*^*2*^ [2]	*Guaman* [24]	*Our data*
**Data source**	CHNC	NRN	VON	BPD Collaborative	NCH
**Number**	2806	866	813	564	485
**Dates**	2010–2015	2011–2015	2018	2015–2019	2014–2020
**Mortality**	6.7%	8%	12.7%	3%	4%
**PMA at D/C (weeks)**	44 [41–50]	*Gr 2* 45 ± 8 *Gr 3* 54 ± 11	*Gr 1/2* 41 [39–44] *Gr 3* 49 [43–53]	–	47 [42–56]
**LOS (days)**	–	–	*Gr 1/2* 103 [84–127] *Gr 3* 163 [122–233]	*Gr 2* 115 [90–146] *Gr 3* 190 [131–309]	125 [90–188]
**Tracheostomy**	7.7%	11.3%	2.2%	8%	10%
**Grade 3 BPD**	40%	29%	8%	24%	23%

Data shown as median [IQR] or %.

*PMA* post-menstrual age, *D/C* discharge.

*LOS* length of stay, *Gr* Grade.

*CHNC* Children’s Hospitals Neonatal Consortium.

*NRN* NICHD neonatal research network.

*VON* Vermont Oxfort Network.

**Table 5:** Comparison of our sub-group that never had a blood gas after 36 weeks PMA to published established BPD cohorts with blood gas sampling specified.

Reference	*Shin* [22]	*Lagatta* [25]	*Our data*
**BPD grade (number)**	Only grade 2 (*n* = 38)	grades 2 and 3 (*n* = 64)	grades 2 and 3 (*n* = 303)
**Frequency of blood gases at or after 36 weeks PMA**	Daily on non-invasive positive pressure and every 2–3 days on lfnc	Routine blood gases at 36 weeks PMA and prior to discharge	None
**LOS (days)**	111 [100–128]	124 [104–143]	109 [81–145]
**PMA at D/C (weeks)**	43 [42–45]	43 [40–46]	45 [41–52]
**Duration of iMV (days)**	44 [27–63]	–	38 [26–66]

Data shown as median [IQR].

*LOS* length of stay, *PMA* post-menstrual age.

*D/C* discharge, *iMV* invasive mechanical ventilation.

## Discussion

In this relatively large cohort of infants with established BPD who were cared for using a guidelines-driven approach that included eliminating routine blood gas measurements, we found that the majority of infants had no blood gases done after admission to the BPD NICU. Our data also suggest that the infants that had blood gases done may have had greater disease severity than did the infants who had no blood gases done after admission to the BPD NICU. It is interesting to note, that even in those sicker infants who had clinically indicated blood gas measurements the median number of blood gases done was only 4 during a relatively long length of stay. Our findings suggest that patients with established severe BPD can be managed without the use of routine blood gas measurements without increased adverse effects when compared to other cohorts. Moreover, the lack of routine blood gas measurements after admission to the BPD NICU in this cohort of infants with established BPD did not seem to impact liberation from respiratory support at least compared to other published cohorts of infants with established BPD. These findings support the notion that infants with established BPD can be effectively managed in the NICU after the diagnosis is made at 36 weeks PMA without routine use of blood gas measurements.

Blood gases are typically used to monitor the efficacy of ventilation in patients with BPD and to guide decisions regarding weaning or escalation of respiratory support. Recent clinical reviews by Abman et al. [16] and Gibbs et al. [26] suggest that routine physical examination, assessment of growth, and tolerance of developmental therapies may be effective in assessing adequacy of ventilation and need for changes in respiratory support in patients with established BPD. The bedside assessment of work of breathing and oxygen saturations provides important information regarding the pulmonary status of patients with established BPD [27]. In our unit, an infant with tachypnea, chest wall retractions, increased work of breathing, and agitation is considered under-supported and changes in respiratory support are made by our multidisciplinary BPD team. The effect of these changes can then be evaluated by assessing for improvements in the patient’s level of comfort, chest rise, FiO_2_, oxygen saturation, growth parameters, and tolerance of developmental therapies. Similarly, weaning of respiratory support can be guided by longitudinal assessments of FiO_2_, growth, and tolerance of developmental therapies. Routine nutrition labs are done in our unit on a weekly to monthly basis depending on patient’s nutritional status. The serum bicarbonate can be used as a surrogate to track changes in the pCO2, however we do not routinely rely on the serum bicarbonate when making changes to respiratory support. Our results also suggest that by eliminating routine blood gas sampling the multidisciplinary care team became comfortable with these non-invasive assessments, which resulted in 62% of our cohort never having a blood gas sampled after admission to the BPD NICU.

Interestingly, there were 18 patients who never had a blood gas after admission to the BPD NICU who underwent tracheostomy placement after admission. The decision to recommend a tracheostomy in an infant with severe BPD is complex and usually involves a continued need for high levels of respiratory support with marginal improvement over time or presence of airway abnormalities [28]. Recently Yallapragada et al. [29] surveyed 31 Children’s Hospitals and found that there are many qualitative indications that inform the decision to recommend a tracheostomy in patients with severe BPD. Interestingly, when asked for relative importance of indications the top 3 were: multiple extubation failures (90%), specific pCO_2_ values (86%), and PMA (82%) [29]. Our study suggests that pCO_2_ may not be a critical component of the decision to recommend tracheostomy, at least in this cohort of sBPD patients. Moreover, trending of FiO2 as well as serum bicarbonate values may provide useful longitudinal assessments of gas exchange in the chronic phase of sBPD.

To our knowledge, this is the first report of infants with established BPD who did not undergo routine blood gas sampling after the diagnosis of severe BPD is made at 36 weeks PMA. It has been reported that in one center all patients with established BPD had at least one blood gas done prior to discharge and that discharge from the NICU required a pCO_2_ < 60 mm Hg [21]. This practice was based on a study that found that the pre-NICU discharge pCO_2_ was associated with adverse post-discharge outcomes, including an increased risk of re-admission [30]. Interestingly, although some practices have been based on this notion, Dawson et al. did not observe that capillary pCO_2_ at 36 weeks PMA or discharge were associated with re-admission in the first year of life [21]. It is also important to remember that there are no widely accepted standards for optimal pCO_2_ levels in infants with BPD [31]. The optimal utility of routine blood gas determinations in established BPD requires further study.

Vinall et al. demonstrated that in a 50-member cohort of former very preterm infants a higher number of tissue-breaking procedures, including heel lances, were associated with lower fractional anisotropy on magnetic resonance imaging, a measure of white matter differentiation, and a lower IQ at a median follow up age of 7 years [32]. A secondary analysis of the Caffeine for Apnea of Prematurity trial data found that BPD was independently associated with increased odds of death or neurodevelopmental impairment at 5 years of age [9]. While the mechanisms underlying the association between BPD and neurodevelopmental impairment remain uncertain [12], there is a clear association between BPD and neurodevelopmental impairment. It is plausible that avoidance of unnecessary painful procedures, including blood gases, in patients with BPD may mitigate the risk and/or severity of neurodevelopmental impairment in early childhood, though additional prospective studies focused on long-term neurodevelopmental outcomes are needed to test this hypothesis.

Our study has important limitations. First, the retrospective, single center design of the study may potentially limit the generalizability of our findings. Second, the specific clinical circumstances surrounding blood gas sampling were not assessed. Thus, our conclusions are limited to routine blood gas sampling, and there are circumstances where clinically indicated blood gas sampling may provide important information to the clinician. Further studies are warranted to examine how to optimize the information obtained by blood gas analysis in concert with clinical assessments to develop interventions that improve outcomes in patients with established severe BPD. Lastly, our study did not examine neurodevelopmental outcomes and given the potential association between repeat blood gas sampling and NDI, future prospective studies are needed to focus on neurodevelopmental outcomes in early childhood in established severe BPD.

In conclusion, we found that patients with established severe BPD can be managed without routine blood gas analyses after 36 weeks PMA. Our results also suggest that the implementation of other measures of adequate respiratory support (FiO_2_, work of breathing, tolerance of therapies) in addition to the elimination of routine blood gas sampling after 36 weeks PMA may decrease overall utilization of blood gases in established severe BPD. Prospective studies are needed to determine the efficacy of alternative assessments of respiratory support across centers and BPD programs.

## Data Availability

The datasets generated during and/or analyzed during the current study are not publicly available due patient privacy but are available from the corresponding author on reasonable request.
